# Discovery of a second citric acid cycle complex

**DOI:** 10.1016/j.heliyon.2023.e15968

**Published:** 2023-05-12

**Authors:** Dirk Roosterman, Graeme Stuart Cottrell

**Affiliations:** a Independent Scholar, Germany; b School of Pharmacy, University of Reading, Reading, RG6 6AP, UK

**Keywords:** Citric acid cycle, Pyruvate dehydrogenase complex, Proton-linked monocarboxylate transporter

## Abstract

Together, Nobel Prize honoured work, mathematics, physics and the laws of nature have drawn a concept of clockwise cycling carboxylic acids in Krebs’ Citric Acid Cycle. A Citric Acid Cycle complex is defined by specific substrate, product and regulation. Recently, the Citric Acid Cycle 1.1 complex was introduced as an NAD^+^-regulated cycle with the substrate, lactic acid and the product, malic acid. Here, we introduce the concept of the Citric Acid Cycle 2.1 complex as an FAD-regulated cycle with the substrate, malic acid and the products, succinic acid or citric acid. The function of the Citric Acid Cycle 2.1 complex is to balance stress situations within the cell. We propose that the biological function of Citric Acid Cycle 2.1 in muscles is to accelerate recovery of ATP; whereas in white tissue adipocytes our testing of the theoretical concept led to the storage of energy as lipids.

## 1 Introduction

Katchalsky and Curran “Living systems are non-equilibration, open systems in which irreversible processes occurring …” [1]. In other words, every breath an organism takes unidirectionally exchanges energy and material with the environment. The 4th law of thermodynamics has to be applied to organisms.

Mainly mathematical approaches were used to transfer non-equilibration thermodynamics (NET) to biology to answer the following questions: How reversibly acting enzymes change into irreversible acting enzymes? and what happens when the product of enzyme A is substrate of enzyme B? [2]. The simplest answers for the questions are: i. an enzyme acts unidirectionally when the concentration of the substrate is infinite or the concentration of the product is zero and ii. a flow of energy and material is sufficient to form ordered structures such as enzyme complexes [3].

Experimental work has also opened a path to transfer NET to biology. Crystal structure analysis of the active site of enzymes revealed that a substrate is stabilized (bound) by the enzyme. Bound in the active site, the substrate is free of water. Thus, in the active site of an enzyme the concentration of substrate/product is infinite/zero because of the absence of a solvent [4]. Enzyme reaction economics strongly suggests that the water-free product is directly transferred to the coupled enzymatic step. Thereby, the hydration energy is saved and transferred to the coupled process. Briefly, direct transfer of a water-free intermediate within an enzyme complex provides an intermediate in infinite concentration and changes a reversible into an irreversible process.

Infinite and zero lie outside the integral formulation of mathematic approaches based on equilibrium thermodynamics. At zero or infinite the physical quantity of concentration [mol/L] is invalid and an equilibration reaction has changed into a unidirectional flow depending on substrate provision. The physical quantity of flow or provision is molecule/time.

The original Citric Acid Cycle (CAC), discovered by H. A. Krebs, shows clockwise cycling of carboxylic acids and the chemical formulae for the ‘burning’ of lactic acid [5,6]. The concept of the original Citric Acid Cycle lies outside an understanding coined by equilibration thermodynamics [7]. Firstly, carboxylic acids rapidly dissociate in water and secondly isolated enzymes reversibly catalyse an equilibrium. The original CAC represents the transfer of NET to the molecular level of biology by showing a unidirectionally acting enzyme complex and direct transfer of water-free intermediates.

Since discovery of the CAC, every single enzymatic reaction has been investigated to confirm the original CAC. However, experimental data not in line with the original concept was assumed to be gospel and thus the original CAC has been morphed into various Krebs or Citrate Cycles. In contrast, experimental data supporting the original concept were often discarded as they did not fit in the Krebs or Citrate Cycles. Thus, the original clockwise cycling wheel of carboxylic acids simultaneously generating energy and biosynthesis had to be discovered for a second time. However, this time together it has been formulated with experimental data accumulated over the past 85 years supporting the original CAC and by using physics; changing the original CAC into a CAC complex to complete the vision of H. A. Krebs [9].

Karpusas and co-workers’ crystal structure analysis proposed a mechanism for the condensation reaction of citrate synthase. In line with the original CAC, the authors discussed oxaloacetic acid as substrate and citric acid as product of citrate synthase [10]. Between 1960 and 1990, L. Reed investigated the enzyme family of α-ketoacid dehydrogenases (2-oxoacid dehydrogenases), such as pyruvate dehydrogenase (PDHc) and α-ketoglutarate dehydrogenase (αKGDH) [11,12]. The catalytic mechanism as well as stoichiometry of each catalysed step dictates pyruvic acid and α-ketoglutaric acid as substrate of PDHc and αKGDH, respectively [11,12]. D. E. Green summarized this as ‘intermediates do not accumulate during normal working of the cyclophorase complex’ indicating that CAC complexes act independently from changes in substrate concentration [13]. In conclusion, Nobel Prize honoured experimental approaches, Nobel Prize honoured mathematics and physics as well as the Laws of Nature confirmed the original concept of the CAC.

Recently, we re-introduced the original CAC in biochemistry and set O. F. Meyerhof's term ‘incomplete burning’ in mechanism [14]. ‘Incomplete burning’ was introduced as delayed recovery of the co-enzyme of malate dehydrogenase (MDH) [9]. We discussed that MDH and respiration complex I must form one complex for direct transfer of water-free NADH-H^+^. Assuming that MDH and respiration complex I cooperate as one complex and share a single molecule NAD^+^, then the activity of MDH depends on recovery of a single NAD^+^ molecule and delayed return of NAD^+^ holds MDH inactive and stops cycling of the CAC complex at MDH. The cease in cycling by delayed recovery is ‘incomplete burning’, allows filling of the cycle with one molecule freshly formed oxaloacetic acid and produces one molecule of clockwise formed malic acid as product of ‘incomplete burning’ of lactic acid.

Investigation of muscle metabolism guided H. A. Krebs to the discovery of the CAC. Working muscles produce significant amounts of cytosolic malate and succinate [15,16]. The introduced mechanism of ‘incomplete burning’ explains increased malate synthesis by an imbalance of the flow of fuel (lactic acid) and oxygen in favour of the fuel [17]. With the CAC 2.1, we introduce the development of the bi-cycle (CAC 1.1 and CAC 2.1 complexes) from Krebs' uni-cycle (CAC).

## 2 2-oxoacid dehydrogenase complexes

The first enzyme of an enzyme complex determines substrate provision (molecule/time) or activity of the complex. In the scientific field of the enzyme family of 2-oxoacid dehydrogenases and proton-linked MCTs, it is well-established that the proton of 2-oxoacid is obligatory for the enzymatic step catalysed by pyruvate dehydrogenase, dihydrolipoyl transacetylase and dihydrolipoyl dehydrogenase and provision of the proton initiates membrane transpocation of the carboxylic acid [18]. Nevertheless, the chemical formula of PDHc catalysed reactions is inconsistently formulated. In scientific publications either pyruvic acid or pyruvate and H^+^ are used in formulae as substrate.

RCOCO_2_H + CoA-SH + DPN → RCO-S-CoA + CO_2_ + DPNH-H^+^ [11]

By setting pyruvate and H^+^ instead of pyruvic acid as substrate it is thus tempting to delete H^+^ from both sides of the chemical formula giving the net reaction below:

RCOCO_2_^-^ + CoA-SH + DPN → RCO-S-CoA + CO_2_ + DPNH + [19]

A chemical formula defining undissociated pyruvic acid is supported by experimental data. G. Brooks laboratory characterized the mitochondrial LDH-h-proton-linked MCT1 complex [20]. Mitochondrial LDH-h catalyses oxidation of lactate to pyruvate and proton-linked MCT1 catalyses formation and membrane transfer of pyruvic acid [21]. PDHc binds tightly to the inner mitochondrial membrane [22]. As mentioned above, a flow of energy and material is sufficient to form ordered structures such as enzyme complexes [3]. Thus, it is the 4th law of thermodynamics guided us to combine experimental data showing formation of pyruvic acid and tightly bound PDHc to the mechanism of direct transfer of water-free pyruvic acid from proton-linked MCT1 to PDHc.

The catalysed net reaction of LDH-h and proton linked MCT1 is:

lactic acid → pyruvic acid + 2H

**Fig. 1:**
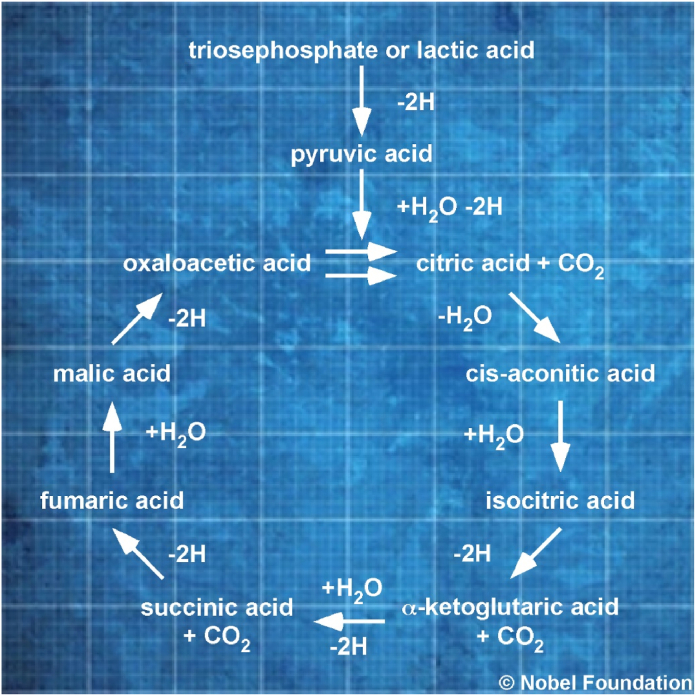
**Krebs' Original Citric Acid Cycle.** This blueprint of Krebs' Citric Acid Cycle is taken from his Nobel Prize lecture, 1953 and was first published 1937 [5,6,8]. Importantly, this degradative unidirectional cycle contains carboxylic acids. ©Nobel Foundation.

H. A. Krebs formulated this chemical formula as first catalysed reaction of the original CAC. The chemical formula is supported by the Laws of Nature, experimental work and NET (Fig. 1).

### 2.1 Transfer of NET to the molecular level of metabolism

At the time Krebs formulated the chemical formula development of NET was in its infancy [23,24]. Formation of one molecule of pyruvic acid from one molecule of pyruvate and one molecule H^+^ results in the production of order. Experimental data has opened a path to transfer production of order to biology. Becker and co-workers demonstrated that plasma membrane located proton-linked MCT1 is functionally linked to carbonic anhydrase II (CAII) [25]. Thus, permanently emitted carbon dioxide/carbonic acid transfers the active proton of carbonic acid to proton-linked MCT1. Transferring the energy of a proton to proton-linked MCT initiates binding of the monocarboxylate and defines the direction of carboxylic acid transport [21]. Briefly, activity and catalytic direction of proton-linked MCT1 is determined by a permanent and unidirectional flow of carbonic acid/carbon dioxide. Proton-linked MCTs and CAII catalyse production of order. In other words, the proton-linked MCT1-CAII complex reverses, leading to energy from outside cell to be stored inside as a cytosolic lactate:pyruvate non-equilibrium.

The first enzymatic steps of original CAC are more precisely formulated as:

i. Lactic acid → pyruvic acid + two hydrogen
ii. Carbonic acid ← carbon dioxide + (water)

A non-equilibrium in favour of lactate is a prerequisite for starting the original CAC. Experimental data leads us assume that mitochondrial proton-linked MCT1 is also in complex with mitochondrial CAII and permanently emitting carbon dioxide/carbonic acid promotes formation and transfer of pyruvic acid [20,26].

The developed concept of proton transport chains opens an understanding to how a single cell simultaneously imports and exports monocarboxylic acids independently from a carboxylate gradient [[27], [28], [29], [30]]. Thus, allowing cells to constantly violate the 2nd law of nature [31].

In contrast to setting pyruvic acid as substrate of PDHc in net chemical formula, setting pyruvate as substrate fails to gain any scientific support. Deleting H^+^ from both sides of the chemical formula is common procedure in chemistry and in the context of a homogenous solution. However, in a homogenous solution the H^+^ has reacted with water. Thus, not H^+^, but H^+^[H_2_O]_n_ is deleted from the chemical formula. A chemical formula illustrating H^+^ ignores the fact that the energy of a blank H^+^ is −262.4 kcal/mol or approximately 35 times the energy freed by hydrolysis of ATP. Moreover, such a chemical formula indicates that the pH does not change and pyruvic acid and NADH-H^+^ share an identical pK_A_ [32]. Briefly, a blank H^+^ does not exist in nature [33]. H^+^ represents the pH and thereby an assumed reaction of H^+^ with water to H^+^[H_2_O]_n_.

Biological organisms are highly organized, where the continuous provision of substrate is obligatory. Considering substrate provision and thereby LDH-h-proton-linked MCT1 as first enzymes of the PDHc, the depicted two H^+^ are in fact a single H^+^. One H^+^ located on opposite sides of a biological membrane, but at two different time points.

Taken together, experimental data demonstrates the production and transfer of pyruvic acid and that pyruvic acid is the substrate for membrane anchored PDHc. Direct transfer of pyruvic acid from proton-linked MCT1 to PDHc maximize acetyl-SCoA synthesis. Direct transfer of water-free intermediates is a huge evolutionary advantage. Any scenarios including the reaction of pyruvic acid with water minimize the efficiency of acetyl-SCoA synthesis and ignores key mechanisms of biology.

For more than a century lactic acid has been a central molecule in metabolism and science. D. E. Green: “In the first, glucose is fermented or split into two molecules lactic acid. In the second, lactic acid is oxidized to carbon dioxide and water by the so-called citric acid cycle discovered by H. A. Krebs of the University of Sheffield.” [34]. O. F. Meyerhof was honoured with the Nobel Prize for “his discovery of the fixed relationship of oxygen and the metabolism of lactic acid”, C. F. and G. T. Cori investigated “glycogen formation from lactic acid”, O. Warburg investigated fermentation of glucose to lactic acid in cancer cell and as discussed the first enzymatic reaction of the original CAC presented by H. A. Krebs at his Nobel Prize lecture shows the oxidation of lactic acid to pyruvic acid [[35], [36], [37]]. More recent experimental work repeated the pioneering work on lactic acid by determining that circulating lactate as main substrate of the TCA cycle [38].

We have transferred NET to the molecular level of metabolism and have summarized that glycolysis and the import of circulating lactic acid combine to create a cytosolic lactate:pyruvate non-equilibrium. NET provides the physics to replace pyruvate with lactate as the central molecule and allows to understand metabolism as the common denominator of biological processes. For example, the mitochondrial LDH-h-proton-linked MCT1 complex feeds the CAC 1.1 complex and PDHc. The competition for the substrate, lactic acid provided a path to solve the riddle of how olanzapine treatment is linked to decreased free fatty acid levels in blood [39]. Olanzapine treatment has been shown to decrease the mRNA levels of both proton-linked MCT1 and LDH-h [40]. Thus, down-regulation of first enzymes of the PDHc quenches acetyl-SCoA production. In compensation, acetyl-SCoA synthesis is shifted to β-oxidation, decreasing free fatty acids.

Up until now, we have described and brought together memory formation, schizophrenia and the beneficial and side effects of the pharmacological treatment of schizophrenia using metabolism [41,42]. We believe that cell surface located heparan sulphate proteoglycans are promising enzymes to add cancer, angiogenesis, inflammation and wound healing to metabolism. It will be of interest to investigate if cell surface proton-linked MCTs and heparan sulphate proteoglycans are functionally linked. If the sulphate proteoglycans create a micro-environment more acid than the surrounding environment this would promote import and restrict export of lactic acid [43].

Taken together, we have started to build up an understanding of biology on the basis of NET. The CAC 1.1 and 2.1 complexes are just the very beginning.

### 2.2 Citric acid cycle complex 2.1

Experimental data has shown that burning of fatty acids requires a ‘sparkle’ of malate [13,44]. The data indicate the existence of a second CAC complex filled with malic acid.

Whereas the CAC 1.1 complex is anchored at pyruvate carboxylase to enable direct transfer of water-free oxaloacetic acid, the CAC 2.1 complex must be anchored at a mitochondrial dicarboxylate carrier (SLC25A10) for direct transfer of malic acid [9]. The mechanism of ‘incomplete burning’ predicts that the cycling of CAC 2.1 complex must be quenched to feed the cycle with malic acid. The first suitable co-enzyme-dependent enzyme able to stop cycling is succinate dehydrogenase (SDH). Thus, quenched recovery of FAD stops cycling, holds MDH in the active (NAD^+^) state and enables filling of the CAC 2.1 complex with malic acid. Halted cycling at SDH triggers a ‘metabolic traffic jam’ and clockwise formed succinic acid is released as product of ‘incomplete burning’ of malic acid into the pool of carboxylates.

The CAC complexes functionally link succinic acid synthesis to exercise. Succinate is the proton-carrier molecule driving ATP synthase as well as a metabolic signalling molecule indicating exercise [16,45,46]. Interestingly, the metabolic signalling molecule, succinic acid provides a third piece of information. Cells export succinic acid not via the exporting transporter such as proton-linked MCT4, but via the importing transporter proton-linked MCT1. Slightly acidic cytosolic pH reverses the catalytic direction of proton-linked MCT1 [16]. Reversing the catalytic direction protects the cells from lactic acid import and further acidification of the cytosol.

In muscles, the CAC 2.1 complex is activated by high lactate levels and triggers the following biosynthetic pathway: lactic acid → CAC 1.1 → malic acid → CAC 2.1 → succinic acid. The leads us to propose that the biological function of this CAC complex is to accelerate the recovery of ATP. During muscle work, enzymes do not catalyse faster in response to increased lactate, it is simply that more CAC 2.1 complexes are activated via malic acid synthesis, which in turn accelerates the recovery of ATP.

## 3 How does olanzapine increase the feeding of the CAC 2.1 complex with malic acid?

Holding SDH in its inactive form (SDH-FAD-H_2_), stops cycling of the CAC 2.1 complex and also holds MDH in its active form (MDH-NAD^+^). The glycerol-3-phosphate shuttle is the cytosolic link to quench FAD recovery. Briefly, the glycerol-3-phosphate shuttle transfers two hydrogens to mitochondrial ubiquinone (Q) forming ubiquinol (QH_2_). SDH also recovers activity via Q. In muscle tissue, it was shown that olanzapine treatment upregulates the enzymes of the glycerol-3-phosphate shuttle: glyceraldehyde-3-phosphate dehydrogenase (GAPDH), cytosolic glycerol-3-phosphate dehydrogenase (GPDHc) and mitochondrial glycerol-3-phosphate dehydrogenase (GPDHm) [40]. This olanzapine-dependent upregulation enhances the glycerol-3-phosphate effect, quenching SDH-FAD recovery and increased feeding of the CAC 2.1 complex with malic acid.

**Fig. 2:**
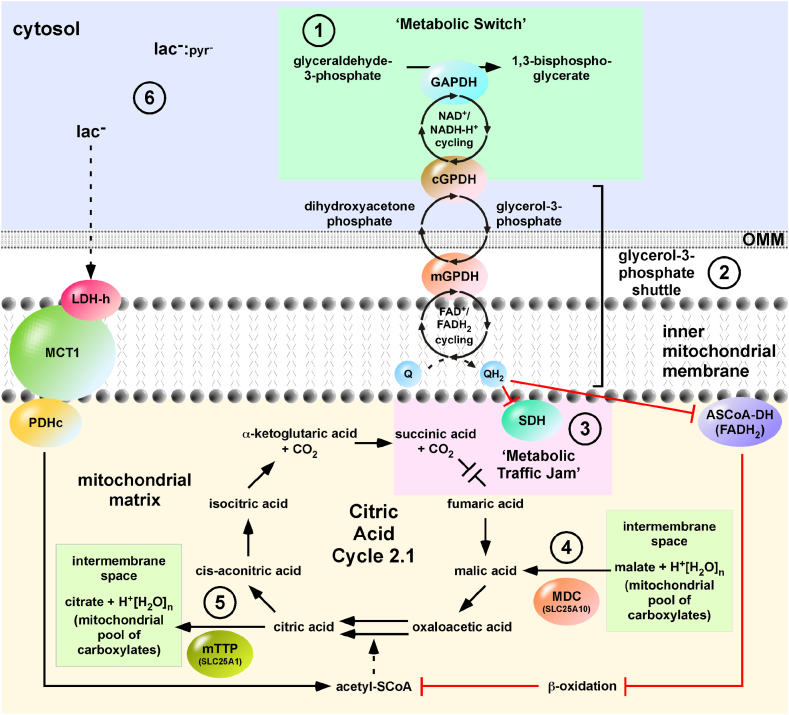
**Regulation of the Citric Acid Cycle 2.1.** (**1**) The regulation of the Citric Acid Cycle 2.1 starts with the ‘metabolic switch’ comprising glyceraldehyde-3-phosphate dehydrogenase (GAPDH) and cytosolic glycerol-3-phosphate dehydrogenase (cGPDH). The enzyme complex ensures direct transfer of water-free NAD^+^/NADH-H^+^ converting glyceraldehyde-3-phosphate to 1,3-bisphosphoglycerate and dihydroxyacetone phosphate to glycerol-3-phosphate. (2) The FAD-dependent mitochondrial form of GPDH (mGPDH) catalyses the reverse reaction, the formation of dihydroxyacetone from glycerol-3-phosphate. This process is often referred to as the glycerol-3-phosphate shuttle. (**3**) FAD-dependent enzymes recover activity by reducing ubiquinone (Q) to ubiquinol (QH_2_). The high levels of generated QH_2_ blocks (red lines) and holds succinate dehydrogenase (SDH) in the reduced (FADH_2_) form, creating a ‘metabolic traffic jam’ at succinic acid in the Citric Acid Cycle 2.1. At acyl-SCoA dehydrogenase (ASCoA-DH), QH_2_ prevents β-oxidation as path for replenishing the mitochondrial acetyl-SCoA pool. Reduced activity at SDH stops cycling and holds malate dehydrogenase (MDH) in its active form. (**4**) The direct anchoring of the Citric Acid Cycle 2.1 to the mitochondrial dicarboxylate carrier (MDC, SLC25A10) facilitates the import of malic acid from mitochondrial pool of carboxylates. (**5**) When cycling reaches citric acid, citric acid is pushed out of the cycle likely due to the blockage of the cycle at IDH. The citric acid is then transported to the intermembrane space by the mitochondrial tricarboxylate transport protein (mTTP, SLC25A1), replenishing the mitochondrial pool of carboxylates. (**6**) The cytosol which has an imbalance of the lac^−^:pyr^−^ ratio in favour of lac-continues to provide lac^−^ as source of acetyl-SCoA via the actions of heart LDH (LDH-h), monocarboxylic acid transporter 1 (MCT1) and pyruvate dehydrogenase complex (PDHc). Dashed lines indicate free diffusion and OMM = outer mitochondrial membrane. (For interpretation of the references to colour in this figure legend, the reader is referred to the Web version of this article.)

An increased malic acid production is obligatory for increased succinic acid or citric acid synthesis. Olanzapine treatment down-regulates proton-linked MCT1 and LDH-h with a dynamic effect on metabolism [40]. The consumption of lactic acid is decreased whereas β-oxidation is more pronounced. This decreased consumption of lactic acid results in the increased availability of the fuel of the CAC 1.1 complex, which through ‘incomplete burning’ promotes malic acid synthesis (Fig. 2).

## 4 Is glycolytically formed lactate stored as lipid?

We tested if white adipose tissue detects and stores excess lactate as lipids. The mechanisms regulating lipid synthesis are unknown. Genetic knock-down of mitochondrial glycerol phosphate dehydrogenase is linked to a 40% reduction in the weight of white adipose tissue [47]. Olanzapine upregulates the enzymes of the glycerol-3-phosphate shuttle [40]. The data indicate that cytosol and Citric Acid Cycle complexes communicate via cytosolic formed glycerol-3-phosphate and balance lipid homeostasis. Cytosolic regulation of SDH-FAD is central in lipid synthesis but does not explain the change in product from succinic acid to citric acid. Investigation of the precise composition of the CAC complexes and PDHc complexes of muscle and white adipose tissue will likely reveal the mechanism behind the change in product. Tentatively, we propose that NAD^+^-regulated CAC 1.1 complex incompletely burns lactic acid to malic acid. The CAC 2.1 complex consumes FAD-dependent malic acid. Citric acid is the product of delayed recovery of NAD^+^-dependent isocitrate dehydrogenase. So, we suggest that in white adipose tissue, pyruvic acid and not lactic acid is the substrate of PDHc or the CAC 2.1 complex. Consuming pyruvic acid instead of lactic acid impacts the regulation of the complexes and one redox unit (i.e., NADH-H^+^) remains in the cytosol to drive synthesis.

## 5 Discussion

H. A. Krebs cites two references in the figure of the original CAC. In 1937, Krebs published that cellular lactate is increased when cells are incubated with pyruvate. The data supported early work on fermentation demonstrating that lactic acid is end-product of glucose fermentation (C_6_H_12_O_6_ = 2× C_3_H_6_O_3_). The original CAC is Krebs theoretical approach to understand how imported and glycolytically formed lactic acid can simultaneously be the substrate of biosynthesis and “burned” (respirated) to CO_2_ and 2H in vivo. (The stoichiometry of the cycle is: Lactic acid (C_3_H_6_O_3_) goes in → three C, three O and three 2H come out with one round of the cycle).

The double chemical reaction arrow between oxaloacetic acid and citric acid is not deducible from lactic acid as substrate (see Krebs original CAC and Fig. 1). Today, it is known that the respiration pathway (-2H and –CO_2_) already starts at the reaction catalysed by pyruvic acid dehydrogenase. The pyruvic acid dehydrogenase complex participates by forming a pool of acetyl-SCoA and is physically separated from CAC complexes. Krebs mixed the two complexes in one theoretical pathway.

The so-called pyruvate dehydrogenase complex (PDHc) does not belong to the original working model, because the respiration products of the substrate pyruvate are one H^+^ short. Thus, 2H changes to 1H^−^. The change from one to two complexes impacts stoichiometry. The discovered biosynthetic pathway is physically attached at oxaloacetic acid synthase (pyruvate carboxylase). Oxaloacetic acid synthase is the first enzyme of the biosynthetic pathway catalysing biosynthesis by adding the respiration product CO_2_ to pyruvic acid. The stoichiometry of the original CAC is corrected for C. Oxaloacetic acid synthase has no dehydrogenase activity. The stoichiometry has to be re-balanced by adding 2H. Thus, oxaloacetic acid synthase adds H_2_CO_3_ or translated into biochemistry: HCO_3_^−^ (bicarbonate) and ATP.

Today, schematics of metabolic pathways are reminiscent of railway maps. All molecules are connected to metabolic pathways. If you point to one molecule on the map, you will discover how that molecule is degraded and synthesized. Such maps give the impression that metabolism is well understood. In 1965, Kornacker and Ball pointed to pyruvate and wrote “Pyruvate enters the mitochondria where it may either be oxidized to acetyl CoA or converted to oxaloacetate by pyruvate carboxylase” and “The acetyl CoA and oxaloacetate produced condense to form citrate which can either be oxidized to CO_2_ or diffuse out of the mitochondria.” [48]. We discussed that in metabolism, dynamic mechanisms decide between “either … or” between ‘complete’ and ‘incomplete burning’. Moreover, anions such as pyruvate not just enter mitochondria. The absence of any scientific standards in the formulated chemical reactions published by Kornacker and Ball created problems and perpetuated myths. Examples of this include the condensing of the *di*-anion oxaloacetate^2−^ with the charge-neutral acetyl-SCoA to yield the *tri*-anion citrate^3−^ and the implicated net reaction of the PDHc catalysed reaction:

CH_3_–C(O)–CO_2_^-^ + HSCoA → CH_3_–CO-SCoA + CO_2_ + H^−^

Pyruvate + Coenzyme A → Acetyl-SCoA + Carbon Dioxide + Hydride

Simple stoichiometry, the basics of nucleophile substitution, referring to the work of H. A. Krebs or referring the characterization of PDHc, would indicate that pyruvic acid is an intermediate of the PDHc [17].

Compared to Kornbacker and Ball's pathway of citrate synthesis from pyruvate, the presented concept of citric acid synthesis by dynamically interacting CAC 1.1 and CAC 2.1 complexes is a more complicated metabolic pathway. Firstly, ‘incomplete burning’ of lactic acid produces malic acid, secondly malic acid is substrate of CAC 2.1 and finally succinic acid/citric acid is product of incomplete burning of malic acid. So why complicate the issue, when simple models of citrate cycles have already provided the impression that metabolism has been well understood for decades? We all understand that modern biology is interested in mechanisms.

However, the well-established metabolic pathways are based on the rationale of equilibration thermodynamics and the premise of ideal distributed systems [49]. In ideal distributed systems only the concentration of substrate and product can act as regulative parameter. This is because the amount of the catalyser (or enzyme) does not change an equilibrium. In ideal distributed systems all acids have reacted with water and membranes that prevent the diffusion of anions such as pyruvate and citrate are absent [48].

Applying NET to the pioneer work on metabolism and splitting the general model of a citrate cycle into two distinct CAC complexes opens a path to integrate all regulatory mechanisms nature has developed. In other words, enzyme complexes dictate the path of the flows of energy and material going to the organism. As discussed, the flow can be dynamically blocked by the quenched recovery of co-enzymes, or reversed by slightly acidic cytosolic pH.

The flow can also be changed. Dehydrogenases form complexes to cycle NAD^+^/NADH [50]. The GAPDH-GPDHc complex and GAPDH-LDH-m complex are both metabolic switch stands involving the direct transfer of NADH-H^+^ [51,52]. The GAPDH-LDH-m complex guides the flow of energy and material to unidirectionally reduces pyruvate to lactate and creates an (internal) cytosolic lactate:pyruvate non-equilibrium. This gradient enables mitochondrial LDH-h to catalyse the opposite reaction: oxidation of lactate to pyruvate. Thus, in one cell, two opposite reactions are simultaneously catalysed by LDH isoforms: pyruvate is forced to be reduced to lactate and a lactate:pyruvate gradient forces the reverse reaction oxidation of lactate to pyruvate [7].

The coupling partners and sub-cellular localization of proton-donor enzymes such as GAPDH and phosphoglycerate kinase (PGK) are regulated by internal and external messengers. These messengers regulate which metabolic pathway is driven by the energy freed by glycolysis [30,53]. Here we set the olanzapine induced imbalance of metabolic homeostasis in context to changes of mRNA levels of metabolic enzymes [40]. Olanzapine and other atypical antipsychotics cause serious metabolic side effects leading to obesity, hyperglycaemia, diabetes and cardiac effects [54,55].

In summary, the construction of a second Citric Acid Cycle (1949–2023) fed with malic acid has taken a bit longer than expected [44]. H. A. Krebs formulated the original Citric Acid Cycle based on his work on lactic acid degradation in whole pigeon muscle. Whereas Kennedy and Lehninger investigated β-oxidation on isolated rat liver mitochondria and transferred their understanding on to Krebs' scientific concept. Kennedy and Lehninger worked in a closed system using the logic of equilibration thermodynamics. H. A. Krebs worked with organs in an open system. Kennedy and Lehninger determined the carboxylates pyr^−^, oxalacetate^2−^, citrate^3−^, and α-ketoglutarate^2-^ and then simply transferred the experimental data and transformed the scientific concept of a Citric Acid Cycle into the all-pervading citrate cycle that is now found in most textbooks and publications. The transformation is exemplified by an excerpt from their article, “condensation of oxalacetate and pyruvate to yield citrate” [44]. Unfortunately, Kennedy and Lehninger failed to determine and transfer protons to their model, neglected to cite a single manuscript of Krebs CAC complex with the substrate lactic acid and thereby failed to recognize and formulate a second CAC complex with the substrate malic acid. Demonstrating that pyruvic acid is consumed by liver mitochondria does not mean that pyruvic acid replaces lactic acid as the primary substrate of muscles. Instead, the data suggest that liver and white adipocytes ‘burn’ pyruvic acid instead of lactic acid. A cytosolic NADH-H^+^ non-equilibrium is created by consuming pyruvic acid required for gluconeogenesis and lipid synthesis.

## Declaration of competing interest

The authors declare that they have no known competing financial interests or personal relationships that could have appeared to influence the work reported in this paper.
